# Diosgenin Targets CaMKK2 to Alleviate Type II Diabetic Nephropathy through Improving Autophagy, Mitophagy and Mitochondrial Dynamics

**DOI:** 10.3390/nu15163554

**Published:** 2023-08-11

**Authors:** Yujie Zhong, Ruyi Jin, Ruilin Luo, Jiayu Liu, Luting Ren, Yinghan Zhang, Zhongguo Shan, Xiaoli Peng

**Affiliations:** 1 College of Food Science and Engineering, Northwest A&F University, Yangling 712100, China; zyj0048@163.com (Y.Z.); jinruyi@nwafu.edu.cn (R.J.); lrl545@nwafu.edu.cn (R.L.); liujiayu1002@nwafu.edu.cn (J.L.); renluting1031@163.com (L.R.); zhangyinghan777@163.com (Y.Z.); shanzhongguo@nwafu.edu.cn (Z.S.); 2 Faculty of Food Science and Engineering, Kunming University of Science and Technology, Kunming 650500, China

**Keywords:** diosgenin, diabetic nephropathy, CaMKK2, autophagy, mitophagy, mitochondrial dynamics

## Abstract

Diabetic nephropathy (DN) is a worldwide health problem with increasing incidence. Diosgenin (DIO) is a natural active ingredient extracted from Chinese yams (*Rhizoma dioscoreae*) with potential antioxidant, anti-inflammatory, and antidiabetic effects. However, the protective effect of DIO on DN is still unclear. The present study explored the mitigating effects and underlying mechanisms of DIO on DN in vivo and in vitro. In the current study, the DN rats were induced by a high-fat diet and streptozotocin and then treated with DIO and metformin (Mef, a positive control) for 8 weeks. The high-glucose (HG)-induced HK-2 cells were treated with DIO for 24 h. The results showed that DIO decreased blood glucose, biomarkers of renal damage, and renal pathological changes with an effect comparable to that of Mef, indicating that DIO is potential active substance to relieve DN. Thus, the protective mechanism of DIO on DN was further explored. Mechanistically, DIO improved autophagy and mitophagy via the regulation of the AMPK-mTOR and PINK1-MFN2-Parkin pathways, respectively. Knockdown of CaMKK2 abolished AMPK-mTOR and PINK1-MFN2-Parkin pathways-mediated autophagy and mitophagy. Mitophagy and mitochondrial dynamics are closely linked physiological processes. DIO also improved mitochondrial dynamics through inhibiting fission-associated proteins (DRP1 and *p*-DRP1) and increasing fusion proteins (MFN1/2 and OPA1). The effects were abolished by CaMKK2 and PINK1 knockdown. In conclusion, DIO ameliorated DN by enhancing autophagy and mitophagy and by improving mitochondrial dynamics in a CaMKK2-dependent manner. PINK1 and MFN2 are proteins that concurrently regulated mitophagy and mitochondrial dynamics.

## 1. Introduction

Diabetic nephropathy (DN) is a predominant microvascular complication of diabetes mellitus (DM), especially type II (T_2_) DM [1,2]. The prevalence of DN in DM patients is about 30–40% and has become the primary cause of end-stage renal disease (ESRD). A recent descriptive cross-sectional study showed that T_2_DM accounted for 35.6% of newly diagnosed ESRD patients [3]. 

Studies have suggested that the defective autophagy is closely related to the progression of DN [4,5]. Autophagy is a process of delivering damaged endogenous or exogenous materials in cells (e.g., organelles and protein aggregates) to lysosomes for degradation [6]. It is indicated that the recovery of autophagy can maintain the balance of the cellular environment, improve renal function, and slow the development of DN, becoming an important target for DN treatment [7]. The AMP-activated protein kinase (AMPK)-mammalian target of rapamycin (mTOR) is a well-established pathway regulating autophagy. AMPK can directly regulate autophagy via inhibiting mTOR activity or phosphorylating the Ser^757^ site of autophagy-related protein 1 (ULK1) [8,9]. Calcium/calmodulin-dependent protein kinase kinase 2 (CaMKK2), upstream of AMPK, could activate AMPK through the phosphorylation at Thr^172^, ultimately stimulating autophagy. Studies have indicated that CaMKK2 induced autophagy through regulating the AMPK-mTOR or AMPK-ULK1 pathways [10,11]. In addition, the inhibition of CaMKK2 could impair autophagy [11].

Mitophagy is a form of autophagy that selectively recycles the damaged mitochondria. The kidney is a highly metabolic organ that requires plenty of mitochondria to regulate energy generation. Mitochondria are dynamic and highly motile organelles rather than static and isolated structures [12]. To maintain the structure and network, mitochondria undergo successive cycles of fission and fusion (mitochondrial dynamics) [13]. Fission is a process to split mitochondria into two, which consists of two types. Fission at the midzone forms new mitochondria, while fission at the periphery produces defective mitochondria that need to be removed by mitophagy [14]. In mammals, the process of mitophagy is mediated by the PTEN-induced putative kinase 1 (PINK1)-Parkin pathway [15]. There is crosstalk between mitophagy and mitochondrial dynamics. A study showed that mitofusin 2 (MFN2), a protein that regulates mitochondrial fusion, mediates the relocation of Parkin to dysfunctional mitochondria, where Parkin binds to MFN2 with the assistance of PINK1 [16]. Meanwhile, during the fusion process, normal mitochondria could combine with damaged mitochondria to exchange their mtDNA and proteins for compensation [17]. A growing amount of evidence suggests that the mitochondrial dynamics disorder, which usually tends to lead to fission, precedes the development of DN [18,19,20]. The defective daughter mitochondria produced by mitochondrial fission need to be removed by mitophagy [14]. However, uncontrolled mitochondrial fission and impaired mitophagy often coexist in DN [21]. In this condition, the damaged mitochondria accumulated in cells will release massive reactive oxygen species and then lead to renal cell apoptosis. Therefore, the improvement of mitochondrial dynamics and mitophagy is a potential target for the treatment of DN.

Diosgenin (DIO) is an important steroidal sapogenin, mainly found in yams, chinaroot greenbrier, and fenugreek. It exhibits antioxidant, anti-inflammatory, and anti-diabetes activities. DIO is the final product of saponins such as dioscin after being metabolized by intestinal microorganisms. Our previous study suggested that dioscin could improve mitophagy and then protect against diabetic kidney damage in rats [22]. However, it is unclear whether the protective effect of dioscin on kidney damage is attributed to its final metabolite product: DIO of gut microbiota. Therefore, it can be hypothesized that DIO, the smallest unit of dioscin, could relieve DN through the induction of autophagy/mitophagy and the improvement of mitochondrial dynamics. CaMKK2/β is considered an upstream kinase of AMPK, which plays a critical role in autophagy [10,11]. Mitophagy is a form of autophagy, and it could interact with mitochondrial dynamics to maintain the mitochondrial function. Thus, we wonder whether CaMKK2 could regulate mitophagy and mitochondrial dynamics. Therefore, the present study investigated whether DIO protected against DN by restoring autophagy, mitophagy, and mitochondrial dynamics in a CaMKK2-dependent manner. The interplay between mitophagy and mitochondrial dynamics was also studied.

## 2. Materials and Methods

### 2.1. Materials and Antibodies

Streptozocin (STZ, CAS#: 18883-66-4, catalog number: S8050) was purchased from Solarbio Science & Technology (Beijing, China). STO-609 (CAS#: 52029-86-4, catalog number: HY-19805) was purchased from MedChemExpress (Monmouth Junction, NJ, USA). Metformin (Mef, CAS#: 1115-70-4, catalog number: B25331) was obtained from Yuanye Bio-Technology (Shanghai, China). 3-Methyladenine (3-MA, CAS#: 5142-23-4, catalog number: ab120841) and Bafilomycin A1 (Baf-A1, CAS#: 88899-55-2, catalog number: ab120497) were purchased from Abcam (St. Louis, MO, USA). Diosgenin (DIO, CAS#: 512-06-1) was purchased from Spring Autumn Biological Engineering (Nanjing, China). Primary antibodies including PINK1 (23274-1-AP), P62 (18420-1-AP), Parkin (14060-1-AP), p70 ribosomal S6 kinase (P70S6K, 14485-1-AP), CaMKK2 (11549-1-AP), MFN2 (12186-1-AP), optic atrophy 1 (OPA1, 27733-1-AP), and mitofusin 1 (MFN1, 13798-1-AP) were purchased from Proteintech (Chicago, IL, USA). Primary antibodies including AMPK (AF6195) and dynamin-related protein 1 (DRP1, AF6735) were purchased from Beyotime (Shanghai, China). Primary antibodies including LC3 (#83506) and *p*-AMPK (phospho-Thr^172^, #2535) were purchased from Cell Signaling Technology (Danvers, MA, USA). *p*-mTOR (Ser^2448^, GTX132803) primary antibody was purchased from GeneTex (San Antonio, TX, USA). Primary antibodies including *p*-P70S6K (Thr^389/412^, #11974) and *p*-DRP1 (Ser^616^, #12749) were purchased from SAB (College Park, CA, USA). 

### 2.2. Animals and Treatments

Male Sprague Dawley rats, 7–8 weeks old, were purchased from Dossy Experimental Animals Co., Ltd. (Chengdu, China). All rats were maintained in controlled conditions with constant 22–25 °C, 60–70% humidity, and a 12 h light/dark cycle. After acclimatization for 1 week, the rats were placed into two groups: the control group (n = 8) and the diabetic group (n = 32). The establishment of diabetic rats refers to our previous article [22]. The rats were administrated with a high-fat diet (HFD) for 4 weeks and then intraperitoneally injected with STZ (35 mg/kg). One week later, rats with random blood glucose >300 mg/dL were separated into 4 groups: DN group (n = 8), DN + DIO (10 mg/kg, n = 8) group, DN + DIO (20 mg/kg, n = 8) group, and DN + Mef (300 mg/kg, n = 8) group (a positive control). DIO and Mef were administered by gavage for 8 weeks, daily. All animal procedures were approved by the Animal Ethics Committee of Northwest A&F University, and Chengdu Dossy Experimental Animals Co., Ltd. (N20071065).

### 2.3. Measurement of Biochemical Index

The method to obtain serum refers to the previous article [22]. The corresponding commercial reagent kits purchased from Nanjing Jiancheng Bio-engineering Institute (Nanjing, China) were used to determine the urea nitrogen and creatinine levels.

### 2.4. Histological Analysis 

The pathological changes of the kidney were measured by Periodic Acid-Schiff (PAS, G1008, Servicebio, Wuhan, China) and hematoxylin (G1004, Servicebio) and eosin (G1001, Servicebio) (H&E) staining. The pathological alterations were assessed under a stereomicroscope (NikonSMZ25, Tokyo, Japan). 

### 2.5. Transmission Electron Microscope (TEM) Analysis

The observation of the ultrastructure of renal tissues was performed by TEM assay as described previously [23]. After being fixed, dehydrated, and penetrated, the samples of kidney tissue were embedded, cut into ~50 nm ultrathin sections, and then collected on a copper grid. Next, the samples were stained with uranyl acetate (15 min) and lead citrate (10 min) in the dark, successively. Finally, the renal structure was observed under the TEM (Tecnai G2 Spirit Bio, FEI System, Hillsborough, OR, USA).

### 2.6. Cell Culture and Treatments

Proximal tubular epithelial (HK-2) cells were cultured in the DMEM media (containing 5.5 mM glucose, Gibco, Waltham, MA, USA). After culturing to 70–80% fusion, the cells were treated with different samples under HG (30 mM) conditions for 24 h, including DIO (1, 2, and 4 µM), DIO (4 µM) + STO-609 (10 µg/mL and 20 µg/mL), DIO (4 µM) + 3-MA (2 mM and 5 mM), and DIO + Baf-A1 (2 nM and 5 nM). 

### 2.7. Measurement of Cell Viability

The MTT assay was used to analyze cell viability. Briefly, the MTT solution (5 mg/mL, Beyotime) of 10 µL was added to the 100 µL medium. After incubating for 4 h at 37 °C, the culture medium was replaced with 150 µL DMSO (GC203002, Servicebio) for a 10 min reaction. Then, the absorbance was measured using a Microplate Reader (Spark, Tecan Austria GmbH, Grödig, Austria) at 490 nm.

### 2.8. Measurement of Cell Apoptosis

The double Annexin V-FITC and PI staining (Beyotime) was used to investigate the degree of cell apoptosis. After being treated as in Section 2.5, the cells were collected after digestion with trypsin (Beyotime) and centrifugation (1000× *g* for 5 min). Subsequently, the collected cells were re-suspended in 195 µL Annexin V-FITC binding solution and then incubated with 5 µL Annexin V-FITC and 10 µL PI for 20 min in the dark. The cell apoptosis rate was measured using a flow cytometer (FACSAria™ III, BD, Franklin Lake, NJ, USA).

### 2.9. Molecular Docking

The CaMKK2 crystal structure was obtained from the Protein Data Bank (PDB) database (PDB code: 5UYJ) and then modified by Autodocktools (version 1.5.621). The structure of DIO was obtained according to the CAS number (512-04-9). Then, the structural energy of DIO was minimized by the chembiodraw 3D module. The protein–ligand docking was performed by AutoDock-Vina (version 1.1.2). The results were processed and visualized using PyMOL (version 2.3.0) and BIOVIA Discovery Studio 2016. The binding affinity of CaMKK2 and DIO is presented as the lowest energy score of the docking simulation.

### 2.10. siRNA Sequence and Transfection

CaMKK2 siRNA, PINK1 siRNA, and MFN2 siRNA were synthesized by Jihua Biology (Beijing, China). The sequences are as follows: Sense: 5′-CCUCUCAUCCUUGAGCAUCCAdTdT-3′; Anti-Sense: UGGAUGCUCAAGGAUGAGAGGdTdT-3′ (CaMKK2 siRNA), Sense: 5′-GGAUGAAGGCAGACAUCAATT-3′; Anti-Sense: 5′-UUGAUGUCUGCCUUCAUCCTT-3′ (PINK1 siRNA), Sense: 5′-GGUUGGACAGUGAGCUCAATT-3′; Anti-Sense: 5′-UUGAGCUCACUGUCCAACCTT-3′ (MFN2 siRNA). The siRNA was transfected using Lipofectamine 2000 (Invitrogen, Carlsbad, CA, USA) for 6 h. Subsequently, the culture medium was changed to low-glucose DMEM containing 10% fetal bovine serum and incubated for 24 h. Finally, the cells were treated as in Section 2.4. 

### 2.11. MitoTracker Red Staining

MitoTracker Red staining was used to observe the mitochondrial morphology. Briefly, the cells were incubated with MitoTracker Red CMXRos (Beyotime, 50 nM) at 37 °C for 20 min after being treated as in Section 2.6. After being washed with fresh culture medium 3 times, the mitochondrial morphology of the cells was observed under Spinning Disk Confocal Microscope (Andorra, UK). 

### 2.12. Immunofluorescence Assay

The immunofluorescence assay for kidney tissue refers to the previous article [22]. Briefly, the sections of kidney tissues were incubated with the primary antibodies (Parkin, LC3, PINK1, and DRP1) at 4 °C for 12 h. Then, they were incubated with the anti-rabbit secondary antibody labeled with Alexa Fluor 488/555 (Beyotime) for 2 h. In vitro, the cells were incubated with primary antibodies including Parkin and PINK1 at 4 °C overnight after being fixed, transparent, and blocked. Subsequently, the cells were incubated with the anti-rabbit secondary antibody labeled with Alexa Fluor 488/555 (Beyotime) for 2 h. DAPI staining and MitoTracker Red staining obtained from Beyotime were used to stain the nuclei and mitochondria of the cells, respectively. The images were obtained using the inverted fluorescence microscope (Lecia DMI8, Weztlar, Germany).

### 2.13. Immunohistochemistry Assay

The kidney sections for immunohistochemistry were prepared as described previously [22]. Then, they were incubated with MFN2 antibody. Subsequently, the renal sections were treated with the secondary antibody labeled with HRP (Beyotime) for 2 h. Then the sections were incubated with DAB staining (Beyotime) and observed under the stereomicroscope (NikonSMZ25, Tokyo, Japan). 

### 2.14. Western Blot Assay

The protein extraction of cells and rat kidneys as well as the western blot assay were performed as described previously [23]. The primary antibodies include LC3, P62, Parkin, PINK1, AMPK, *p*-AMPK, *p*-mTOR, P70S6K, *p*-P70S6K, CaMKK2, DRP1, FIS1, *p*-DRP1, MFN1, MFN2, OPA1, ACTB, and GAPDH. 

### 2.15. Statistical Analysis

All data are expressed as the mean ± SD. One-way factorial analysis of variance (ANOVA) was used to calculate significant differences between groups, followed by Duncan’s multiple range test using SPSS 20.0. *, significantly different from the control group; #, significantly different from the DN or HG group; ^, significantly different from the HG+DIO group. *, #, and ^ *p* < 0.05; **, ##, and ^^, *p* < 0.01. 

## 3. Results

### 3.1. DIO Relieved Diabetic Nephropathy in Rats

**Figure 1:**
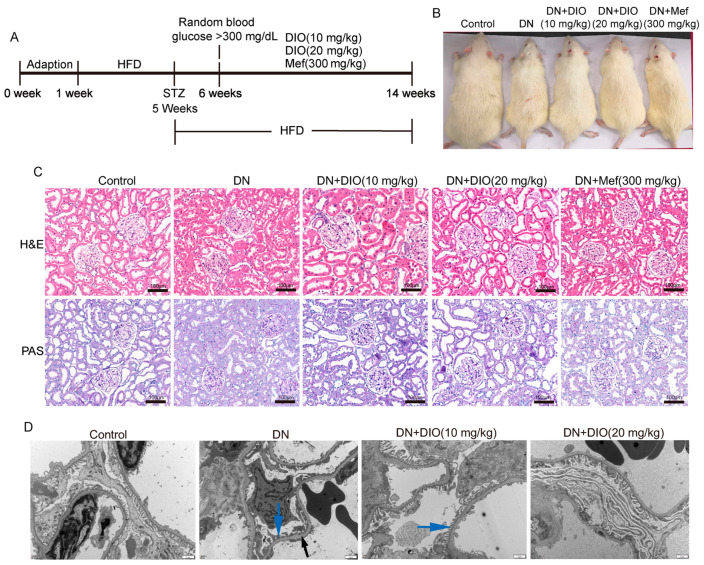
DIO relieved DN in rats induced by HFD+STZ. (**A**) Animal experiment diagram. (**B**) The picture of rats at the end of the experiment. (**C**) The representative images of H&E and PAS staining of the kidney, scale bars = 100 µm. (**D**) The representative TEM images of the glomerulus (glomerular basement membrane, black arrow; Podocyte, blue arrow), scale bars = 1 µm. n = 6 for each experiment.

To investigate the protective effect of DIO on DN, the fasting blood glucose, kidney/body weight, renal function markers, and renal pathology were measured. Figure 1A,B are the animal experiment diagram and the rat picture at the end of the experiment. As shown in Figure 1B, DIO and Mef improved the decrease in body weight in DN rats. The fasting glucose in DN rats increased greatly; after DIO and Mef treatment, it decreased significantly (Table 1). DIO and Mef also decreased nitrogen and creatinine levels but had no significant effect on the kidney index (Table 1). H&E and PAS staining showed mesangial matrix deposition and thickening of the tubular and glomerular basement membranes in DN rats (Figure 1C). The TEM result also showed the thickening of the glomerular basement membrane and the loss of podocytes in DN rats (Figure 1D). These pathological changes were ameliorated by DIO and Mef.

### 3.2. DIO Improved Autophagy and Mitophagy in DN Rats

**Figure 2:**
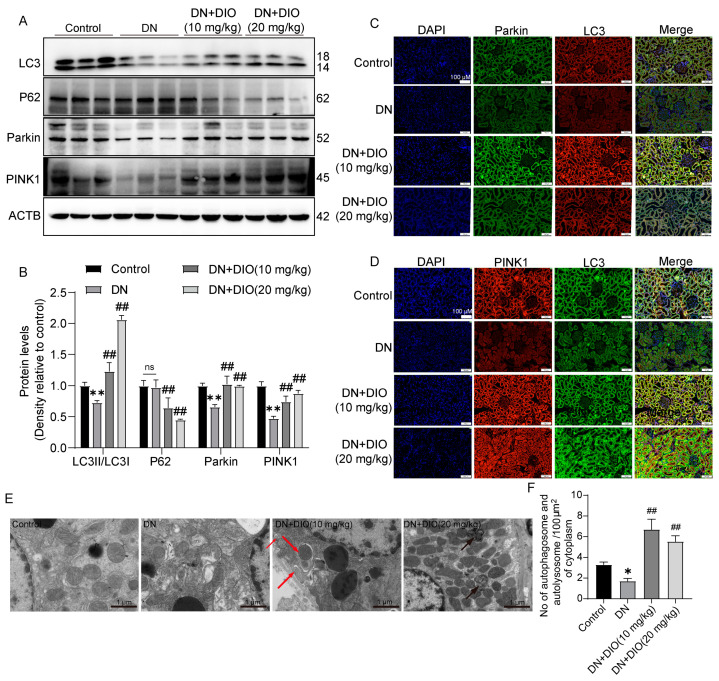
DIO restored autophagy and mitophagy in DN rats. (**A**) The representative images of LC3, P62, Parkin, PINK1, and ACTB immunoblotting in rat kidneys. (**B**) Quantification of LC3II/LC3I, P62, Parkin, and PINK1 protein expressions. (**C**) Fluorescence colocalization of LC3 and Parkin in rat kidneys, scale bars = 100 µm. (**D**) Fluorescence colocalization of LC3 and PINK1 in rat kidneys, scale bars = 100 µm. (**E**) The representative TEM images of mitophagy and autophagy in rat kidneys, scale bars = 1 µm. (**F**) The quantification of autophagosomes and autolysosomes in each 100 µm^2^ of cytoplasm. Data are presented as mean ± SD, n = 6 for each experiment. * *p* < 0.05 and ** *p* < 0.01, significantly different compared to the control group; ^##^ *p* < 0.01, significantly different compared to the DN group; ns, no significance.

Increasing evidence has indicated that autophagy and mitophagy are inhibited under DN conditions. In the present study, the downregulation of LC3II/LC3I was restored by DIO. In addition, DIO decreased P62 expression significantly (Figure 2A,B). Mitophagy is one of the forms of autophagy, which is regulated by Parkin and PINK1. DIO also relieved the downregulation of mitophagy-mediated proteins (Parkin and PINK1) in DN rats (Figure 2A,B and Figure S1B,C). Furthermore, the colocalization of PINK1 with LC3 and Parkin with LC3 was enhanced by DIO (Figure 2C,D). Moreover, autophagosome-vesicle-coated mitochondria or other cellular components (red arrow) and autolysosome structures remaining after autophagosome degradation by lysosomes (black arrows) were also observed in DIO-treated DN rats (Figure 2E). These data suggest that autophagy, autophagy flux, and mitophagy were enhanced by DIO in DN rat kidneys.

### 3.3. DIO Increased Autophagy and Mitophagy in HK-2 Cells Treated with HG 

**Figure 3:**
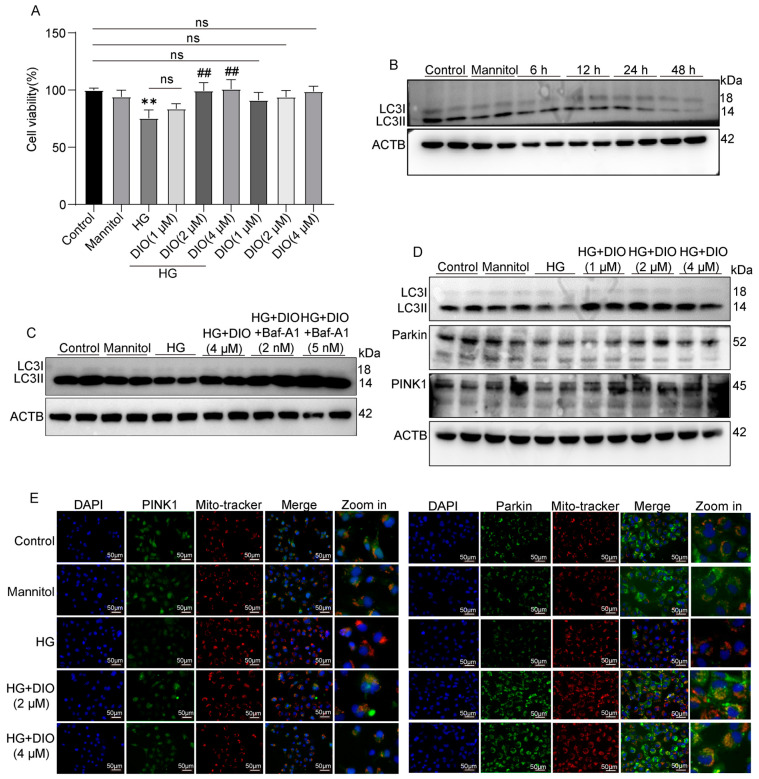
DIO induced autophagy and mitophagy in HK-2 cells exposed to HG. (**A**) Cell viability of DIO-treated HK-2 cells with or without HG (30 mM) for 24 h. (**B**) The representative images of LC3 and ACTB immunoblotting of HG (30 mM) treatment for 6, 12, 24, and 48 h. (**C**) The representative images of LC3 and ACTB immunoblotting of HG+DIO+Baf-A1 treatment. (**D**) The representative images of LC3, Parkin, PINK1, and ACTB immunoblotting of HG+DIO treatment. (**E**) The mitochondrial localization of PINK1 and Parkin, scale bars = 50 µm. Data are expressed as mean ± SD, n = 6 for each experiment. ** *p* < 0.01, significantly different compared to the control group; ^##^ *p* < 0.01, significantly different compared to the HG group; ns, no significance.

To further evidence the recovery effect of DIO on autophagy and mitophagy, we conducted in vitro experiments using HK-2 cells. Compared to the HG group, DIO, especially at 4 µM, increased cell viability greatly (Figure 3A). The expression of LC3II/LC3I was downregulated significantly in cells exposed to HG for 6, 12, 24, and 48 h (Figure 3B and Figure S2A). After DIO treatment, the expression of LC3II/LC3I was upregulated (Figure 3D and Figure S2B). Autophagy flux can be measured by comparing the amount of LC3II in cells treated with and without the autophagy lysosomal inhibitor [24]. Our results showed that treatment with Baf-A1 (2 µM), an agent blocking the fusion of autophagosomes with lysosomes, further increased LC3II/LC3I expression, which indicated that DIO increased autophagy flux (Figure 3C and Figure S2C). Consistent with in vivo studies, DIO relieved the downregulation of Parkin and PINK1 in HG-treated HK-2 cells (Figure 3D and Figure S2B,D,E). Immunofluorescence also suggested that DIO increased the mitochondrial localization of PINK1 and Parkin (Figure 3E). Treatment with autophagy inhibitor 3-methyladenine (3-MA, 5 µM) downregulated LC3II/LC3I expression efficiently (Figure S2F). Meanwhile, compared to the DIO treatment group, the cell viability was greatly decreased after treatment with 3-MA (Figure S2G). These results suggested that DIO induced autophagy and mitophagy in HG-treated HK-2 cells, which could protect against cell injury.

### 3.4. DIO Enhanced Mitophagy through the PINK1-MFN2-Parkin Pathway

**Figure 4:**
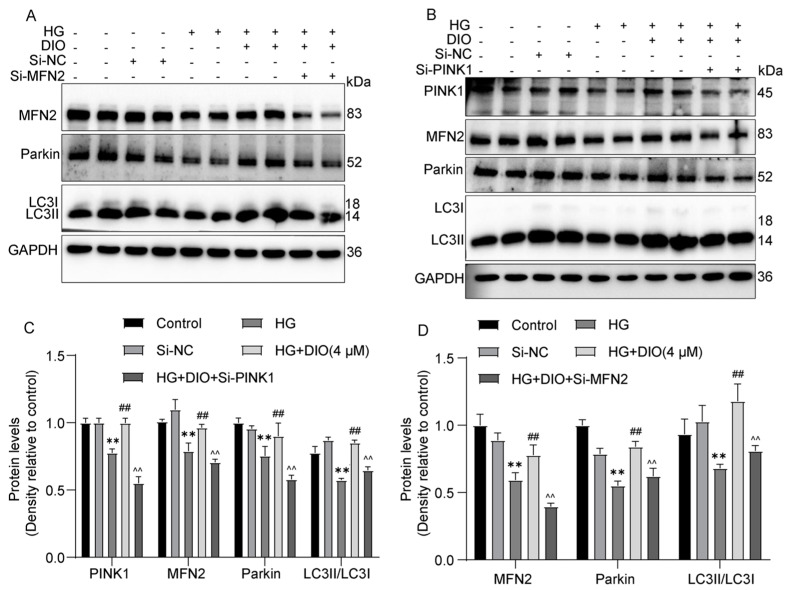
DIO enhanced mitophagy by regulating the PINK1-MFN2-Parkin pathway. (**A**) The representative images of MFN2, Parkin, LC3, and GAPDH immunoblotting in HK-2 cells with MFN2 knockdown. (**B**) The representative images of PINK1, MFN2, Parkin, LC3, and GAPDH immunoblotting in HK-2 cells with PINK1 knockdown. (**C**) Quantification of PINK1, MFN2, Parkin, and LC3 protein expressions in HK-2 cells with PINK1 knockdown. (**D**) Quantification of MFN2, Parkin, and LC3 protein expressions in HK-2 cells with MFN2 knockdown. Data are expressed as mean ± SD, n = 6 for each experiment. ** *p* < 0.01, significantly different compared to the control group; ^##^ *p* < 0.01, significantly different compared to the HG group; ^^ *p* < 0.01 significantly different compared to the HG+DIO group.

MFN2 plays a critical role in mitophagy induction, and PINK1 could activate MFN2 and then promote the translocation of Parkin to damaged mitochondria. As shown in Figure 4A,D, compared to the DIO treatment group, MFN2 siRNA decreased Parkin and LC3II/LC3I expressions. Knockdown of PINK1 abolished the enhanced expressions of MFN2, Parkin1, and LC3II/LC3I induced by DIO (Figure 4B,C). These results suggest that DIO induced mitophagy via regulating the PINK1-MFN2-Parkin pathway. The interference efficiency of PINK1 and MFN2 is shown in Figure S5C,D,G,H.

### 3.5. DIO Activated Autophagy via the AMPK-mTOR Pathway and Enhanced CaMKK2 Expression 

**Figure 5:**
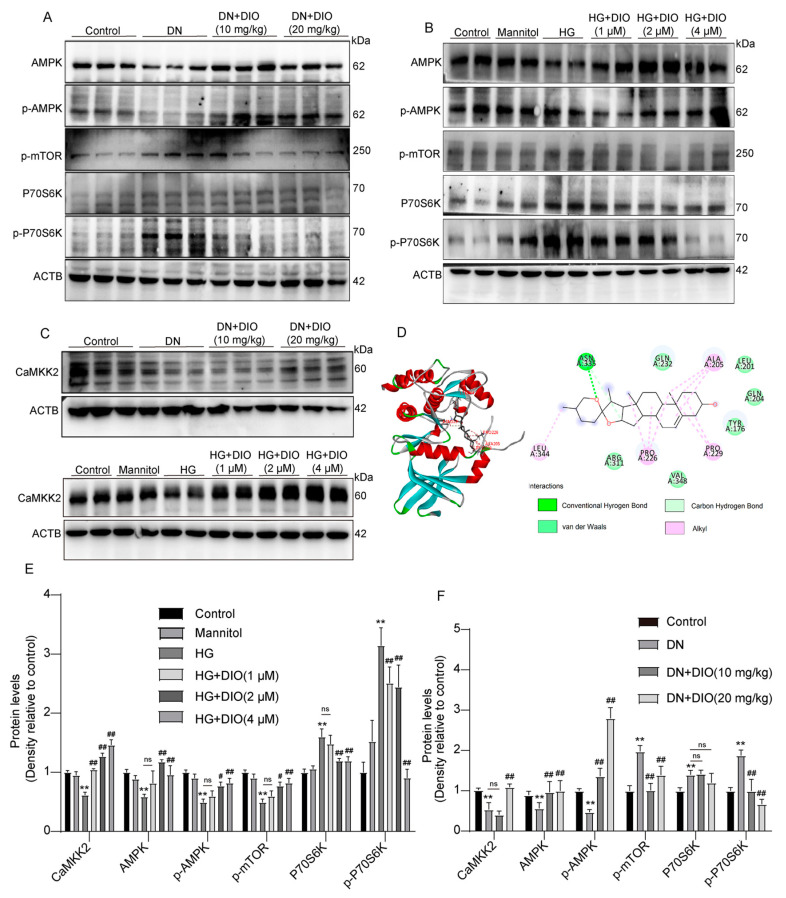
DIO activated the AMPK-mTOR pathway and enhanced CaMKK2 expression in DN rats and HG-treated HK-2 cells. (**A**) The representative images of AMPK, *p*-AMPK, *p*-mTOR, P70S6K, *p*-P70S6K, and ACTB immunoblotting in rat kidneys. (**B**) The representative images of AMPK, *p*-AMPK, *p*-mTOR, P70S6K, *p*-P70S6K, and ACTB immunoblotting in HK-2 cells. (**C**) The representative images of CaMKK2 and ACTB immunoblotting in rat kidneys and HK-2 cells. (**D**) Docking simulation of DIO to the active site of CaMKK2 (PDB code: 5UYJ). (**E**) Quantification of CaMKK2, AMPK, *p*-AMPK, *p*-mTOR, P70S6K, and *p*-P70S6K protein expressions in HK-2 cells. (**F**) Quantification of CaMKK2, AMPK, *p*-AMPK, *p*-mTOR, P70S6K, and *p*-P70S6K protein expressions in DN rats. Data are expressed as mean ± SD, n = 6 for each experiment. ** *p* < 0.01, significantly different compared to the control group; ^#^ *p* < 0.05 and ^##^ *p* < 0.01, significantly different compared to the DN or HG group; ns, no significance.

The AMPK-mTOR pathway plays an important role in regulating energy homeostasis and autophagy. To investigate whether DIO could activate the AMPK-mTOR pathway, the expressions of AMPK, *p*-AMPK, *p*-mTOR, P70S6K, and *p*-P70S6K were measured. As shown in Figure 5A,F, AMPK and *p*-AMPK were downregulated remarkably in DN rats, while *p*-mTOR, P70S6K, and *p*-P70S6K expressions were upregulated. The administration of DIO to DN rats increased AMPK and *p*-AMPK expressions but decreased the expressions of *p*-mTOR, P70S6K, and *p*-P70S6K. Consistent with the results obtained in rats, DIO increased AMPK and *p*-AMPK expressions but decreased *p*-mTOR, P70S6K, and *p*-P70S6K expressions in HG-treated HK-2 cells (Figure 5B,E). CaMKK2 is upstream of AMPK. As shown in Figure 5C–F, the downregulation of CaMKK2 was restored by DIO in vivo and in vitro. Moreover, the results of molecular docking suggested that DIO could interact with CaMKK2 (binding affinity: −8.7 kcal/mol). The pharmacophore analysis revealed that DIO interacted with CaMKK2 by forming a hydrogen bond and a carbon–hydrogen bond with ASNA: 335 residues, several Alkyl with residues of LEUA: 344, PBOA: 226, PBOA: 299, and ALAA: 205, and 6 Van der Waals (Figure 5D). 

### 3.6. DIO Induced Autophagy and Mitophagy in a CaMKK2-Dependent Manner

**Figure 6:**
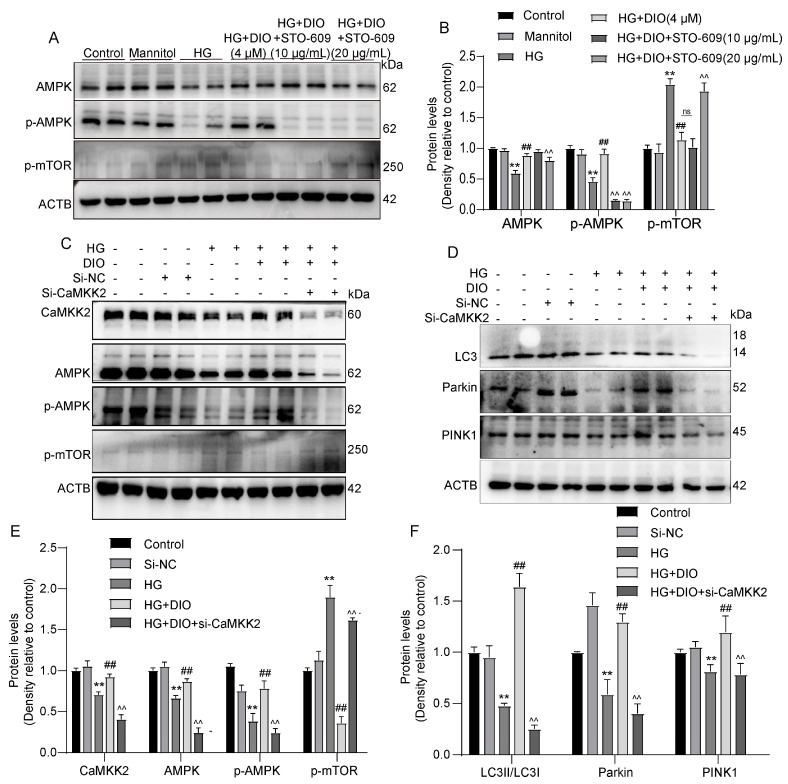
Inhibition of CaMKK2 abolished AMPK-mTOR-mediated autophagy and PINK1–Parkin-mediated mitophagy in HK-2 cells. (**A**) The representative images of AMPK, *p*-AMPK, *p*-mTOR, and ACTB immunoblotting in HK-2 cells treated with STO-609. (**B**) Quantification of AMPK, *p*-AMPK, and *p*-mTOR protein expressions in HK-2 cells treated with STO-609. (**C**) The representative images of CaMKK2, AMPK, *p*-AMPK, *p*-mTOR, and ACTB immunoblotting in HK-2 cells with CaMKK2 knockdown. (**D**) The representative images of LC3, Parkin, PINK1, and ACTB immunoblotting in HK-2 cells with CaMKK2 knockdown. (**E**) Quantification of CaMKK2, AMPK, *p*-AMPK, and *p*-mTOR protein expressions in HK-2 cells with CaMKK2 knockdown. (**F**) Quantification of LC3, Parkin, and PINK1 protein expressions in HK-2 cells with CaMKK2 knockdown. Data are expressed as mean ± SD, n = 6 for each experiment. ** *p <* 0.01, significantly different compared to the control group; ^##^ *p <* 0.01, significantly different compared to the HG group; ^^ *p <* 0.01, significantly different compared to the HG+DIO group; ns, no significance.

**Figure 7:**
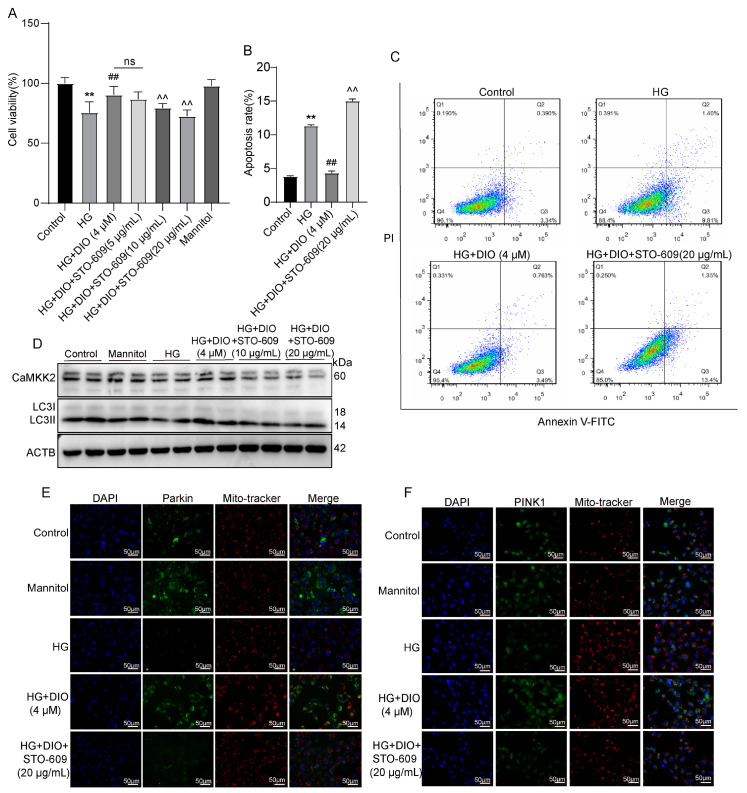
Inhibition of CaMKK2 abolished autophagy and mitophagy induced by DIO in HK-2 cells. (**A**) Cell viability. (**B**) Quantification of apoptosis ratio. (**C**) Cell apoptosis detected by flow cytometry. (**D**) The representative images of CaMKK2, LC3, and ACTB immunoblotting. (**E**,**F**) Mitochondrial localization of PINK1 and Parkin. Data are expressed as mean ± SD, n = 6 for each experiment. ** *p <* 0.01, significantly different compared to the control group; ^##^ *p <* 0.01, significantly different compared to the HG group; ^^ *p <* 0.01, significantly different compared to the HG+DIO group; ns, no significance.

To further investigate whether CaMKK2 induces autophagy through regulating the AMPK-mTOR pathway, specific CaMKK2-inhibitor STO-609 and CaMKK2 siRNA were introduced. The interference efficiency of CaMKK2 is shown in Figure S5B,F. As shown in Figure 6A–D and Figure S3, compared to the DIO treatment group, STO-609 decreased cell viability, increased cell apoptosis, and downregulated LC3II/LC3I, Parkin, and PINK1 expressions. The increased localizations of Parkin and PINK1 on mitochondria induced by DIO were also inhibited by STO-609 (Figure 6E,F). The restored expressions of AMPK and *p*-AMPK by DIO were abolished by STO-609 and CaMKK2 siRNA (Figure 7A–C,E). DIO inhibited the upregulation of *p*-mTOR in HG-treated HK-2 cells, which was abolished by the treatment of STO-609 and CaMKK2 siRNA (Figure 7A–C,E). In addition, the upregulation of LC3, Parkin, and PINK1 in the DIO treatment group was partially abolished by CaMKK2 siRNA transfection (Figure 7D,F). These data suggest that DIO restored autophagy via the regulation of the CaMKK2-AMPK-mTOR pathway and mitophagy via the modulation of the CaMKK2-mediated PINK1-Parkin pathway in HG-treated HK-2 cells.

### 3.7. DIO Ameliorated Mitochondrial Dynamics In Vivo and In Vitro

**Figure 8:**
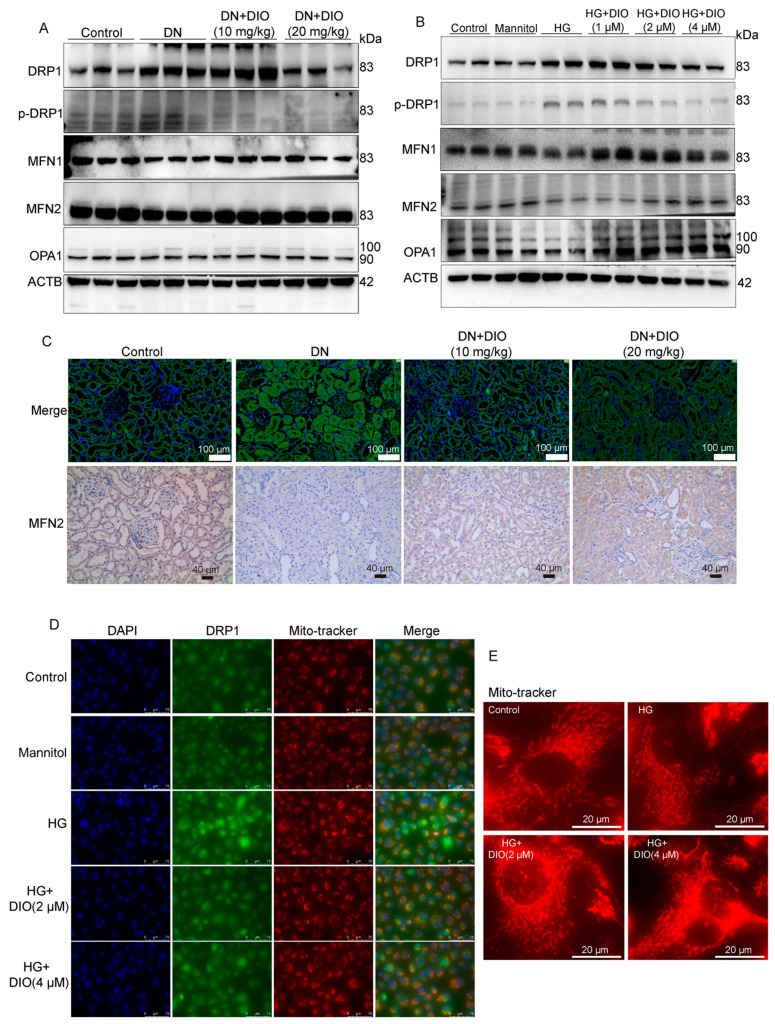
DIO improved mitochondrial dynamics in DN rats and HK-2 cells exposed to HG. (**A**) The representative images of DRP1, *p*-DRP1, MFN1, MFN2, OPA1, and ACTB immunoblotting in rat kidneys. (**B**) The representative images of DRP1, *p*-DRP1, MFN1, MFN2, OPA1, and ACTB immunoblotting in HK-2 cells. (**C**) The representative images of DRP1 immunofluorescence (scale bars = 50 µm) and MFN2 immunohistochemistry (scale bars = 40 µm) in rat kidneys. (**D**) The representative images of DRP1 immunofluorescence in HK-2 cells, scale bars = 75 µm. (**E**) MitoTracker staining (mitochondrial morphology) in HK-2 cells. Data are expressed as mean ± SD, n = 6 for each experiment.

Mitochondrial dynamics (fission and fusion) and mitophagy belong to mitochondrial quality control and are two closely related processes. In Figure 8A and Figure S4C, the increase in mitochondrial fission proteins DRP1 and *p*-DRP1 in DN rats was reversed by DIO treatment. The immunofluorescence results also showed that DIO downregulated DRP1 expression noticeably (Figure 8C and Figure S4B). MFN1/2 and OPA1 are proteins regulating the fusion of mitochondrial membranes. As shown in Figure 8A,C and Figure S4C, DIO upregulated MFN1/2 expression but had no significant effect on OPA1 expression. Similar results were obtained in HK-2 cells. DIO downregulated DRP1/*p*-DRP1 expression and restored MFN1/2 expression (Figure 8B and Figure S4A). In addition, there was a decrease in OPA1 expression in HG-treated cells, and DIO restored it (Figure 8B and Figure S4A). Additionally, the increased mitochondrial localization of DRP1 was inhibited by DIO (Figure 8D). Moreover, mitochondrial fragmentation was observed in HG-treated HK-2 cells, and DIO improved it (Figure 8E). These results confirmed that DIO relieved mitochondrial dynamics disorder. 

### 3.8. Mitochondrial Dynamics Are Partially Regulated by CaMKK2 and PINK1

**Figure 9:**
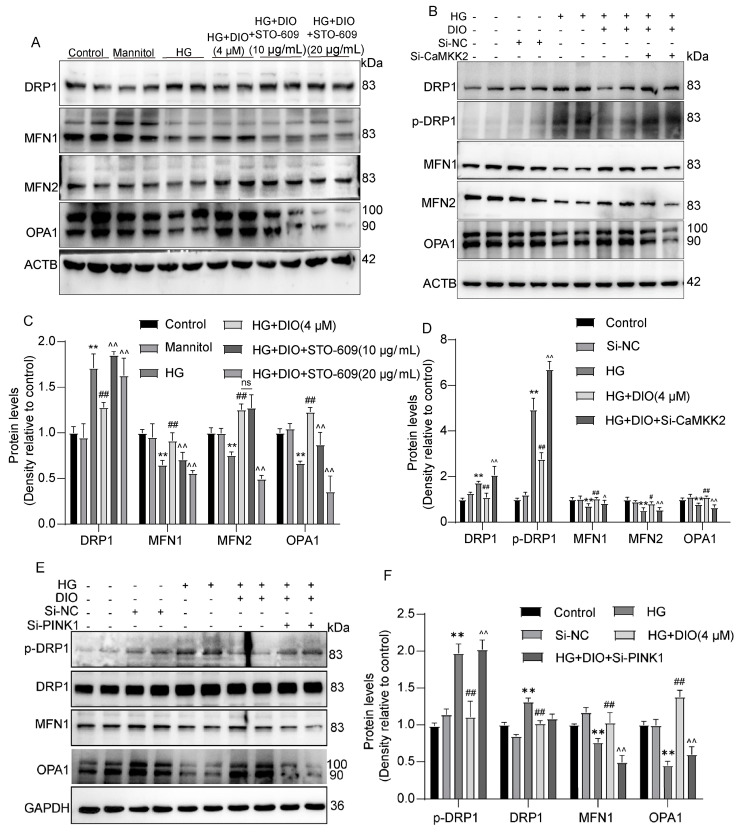
Mitochondrial dynamics are partially regulated by CaMKK2 and PINK1. (**A**) The representative images of DRP1, MFN1, MFN2, OPA1, and ACTB immunoblotting in HK-2 cells treated with STO-609. (**B**) The representative images of DRP1, *p*-DRP1, MFN1, MFN2, OPA1, and ACTB immunoblotting in HK-2 cells with CaMKK2 knockdown. (**C**) Quantification of DRP1, MFN1, MFN2, and OPA1 protein expressions in HK-2 cells treated with STO-609. (**D**) Quantification of DRP1, *p*-DRP1, MFN1, MFN2, and OPA1 protein expressions in HK-2 cells with CaMKK2 knockdown. (**E**) The representative images of DRP1, *p*-DRP1, MFN1, OPA1, and GAPDH immunoblotting in HK-2 cells with PINK1 knockdown. (**F**) Quantification of DRP1, *p*-DRP1, MFN1, and OPA1 protein expressions in HK-2 cells with PINK1 knockdown. Data are expressed as mean ± SD, n = 6 for each experiment. ** *p <* 0.01, significantly different compared to the control group; ^#^ *p <* 0.05 and ^##^ *p <* 0.01, significantly different compared to the HG group; ^ *p <* 0.05 and ^^ *p <* 0.01, significantly different compared to the HG+DIO group; ns, no significance.

Since mitophagy and mitochondrial dynamics are two related processes and CaMKK2/PINK1 could regulate mitophagy, we further explored whether CaMKK2 and PINK1 could modulate mitochondrial dynamics. As shown in Figure 9A–D, the inhibitory effect of DIO on DRP1 and *p*-DRP1 expressions was abolished by STO-6 and CaMKK2 siRNA. In addition, STO-6 and CaMKK2 siRNA abolished the restorative effect of DIO on MFN1, MFN2, and OPA1 expressions (Figure 9A–D). Furthermore, as shown in Figure 9E,F, PINK1 knockdown abolished the ameliorative effect of DIO on mitochondrial dynamics, evidenced by the upregulation of *p*-DRP1 and the downregulation of MFN1, MFN2, and OPA1. These results suggest that mitochondrial dynamics are partially regulated by CaMKK2 and PINK1, which may be due to the inhibition of mitophagy. 

## 4. Discussion

DN is one of the major complications of DM, affecting 30–40% of diabetic patients. Due to the side effects of conventional drugs, various natural substances have been explored to protect against DN such as (poly)phenols, resveratrol, mangiferin, apigenin, etc. [25,26,27,28]. The present study confirmed that DIO could prevent renal damage by improving mitochondrial dynamics and enhancing autophagy and mitophagy via a CaMKK2-dependent manner, in which MFN2 and PINK1 are two proteins linking mitophagy and mitochondrial dynamics. 

Firstly, the elevations of fasting blood glucose and biomarkers of renal damage in DN rats were inhibited by DIO. H&E and PAS staining as well as TEM ultrastructure showed that DIO ameliorated mesangial matrix deposition and the thickening of renal tubular and glomerular basement membranes in DN rats. 

Impaired autophagy leads to the development of DN because of the accumulation of damaged organelles and protein aggregates. Studies have suggested that autophagy induction can protect against DN. For example, Astragaloside IV prevented the progression of DN by enhancing AMPK-promoted autophagy [29]. T-AUCB increased autophagic flux and then improved mitochondrial function and ER stress in HG-treated HK-2 cells and db/db mice [30]. In the current study, DIO restored autophagy and autophagy flux in DN rats. AMPK is a vital molecule in the regulation of energy metabolism, which plays an indispensable role in diabetes and other metabolic-related diseases [31]. It can modulate the process of autophagy to achieve cellular homeostasis by inhibiting the mTOR-P70S6K pathway [32]. Ca^2+^/calmodulin-dependent protein kinase kinase 2 or β (CaMKK2/β) is recognized as an upstream kinase that activates AMPK through phosphorylation at Thr^172^. Further studies established an important role of the CaMKKβ/AMPK axis in promoting autophagy [10,33]. Our study showed that DIO restored CaMKK2 and AMPK expressions, while inhibiting *p*-mTOR and *p*-P70S6K expressions in rats. 

Mitophagy is a process that targets the removal of damaged mitochondria, which is mediated by the PINK1-Parkin pathway [34]. When mitochondria are damaged, PINK1 is recruited to the mitochondrial outer membrane and serves as a molecular signal to recruit Parkin [35]. Then, Parkin polyubiquitinates specific proteins on the outer mitochondrial membrane to form ubiquitin chains, leading to the recruitment of autophagosomal microtubule-associated proteins including LC3 [36,37]. Our study showed that DIO increased PINK1 and Parkin expressions as well as the colocalizations of PINK1 and Parkin with LC3 in rat kidneys. The number of mitochondria in renal tubules is much higher than that in glomerular cells because renal tubules need to produce a large amount of energy to fulfill their high energy demand. In addition, various studies showed that hyperglycemia could directly damages renal tubules, resulting in mitochondria dysfunction, which is a prime mover in DN [38]. Therefore, we chose HK-2 cells as a representative cell model to further investigate the mechanism of the improvement of autophagy and mitophagy by DIO. The results showed that PINK1 and Parkin expressions as well as the mitochondrial localization of PINK1 and Parkin increased greatly in DIO-treated HK-2 cells. Although a study suggested that AMPK can regulate autophagy and mitophagy, as an upstream of AMPK, whether CaMKK2 regulates autophagy and mitophagy is not clear. Thus, the regulatory effect of CaMKK2 on autophagy and mitophagy was also studied in the present study using HK-2 cells. Our results showed that the inhibition or knockdown of CaMKK2 suppressed autophagy by inhibiting the AMPK-mTOR pathway. In addition, the CaMKK2 inhibitor or CaMKK2 Si-RNA inhibited the overexpression of PINK1 and Parkin as well as the mitochondrial localization of PINK1 and Parkin induced by DIO, suggesting that CaMKK2 could regulate mitophagy via the PINK1-Parkin pathway. 

Mitochondria are dynamic organelles that maintain their structure and network through continuous fission and fusion. The fusion of the outer and inner mitochondrial membranes is regulated by MFN1/2 and OPA1, respectively [39]. Mitochondrial fission is regulated by DRP1 and its receptor, FIS1. Of note, the mitochondrial fragmentation caused by excessive mitochondrial fission or impaired mitochondrial fusion will lead to cell apoptosis mediated by mitochondrial pathways [40]. Studies have suggested that excessive mitochondrial fission and fragmentation were observed in the DN progress [18,41,42]. In the present study, disturbances of mitochondrial dynamics tending to fission were observed in DN rat kidneys. The same results were observed in HG-treated HK-2 cells. Meanwhile, DIO treatment inhibited mitochondrial fission and restored mitochondrial fusion via inhibiting DRP1 and *p*-DRP1 expressions and enhancing MFN1, MFN2, and OPA1 expressions. In addition, DIO suppressed the mitochondrial localization of DRP1 in HG-treated HK-2 cells. Since mitophagy and mitochondrial dynamics are closely related cellular physiological processes and CaMKK2 could regulate mitophagy, whether CaMKK2 could regulate mitochondrial dynamics was studied in HK-2 cells. Our results showed that CaMKK2 siRNA abolished the inhibitory effect of DIO on DRP1 and *p*-DRP1 expressions and the restorative effect of DIO on MFN1/2 and OPA1 expressions, suggesting that CaMKK2 could regulate mitochondrial dynamics.

Mitochondrial dynamics and mitophagy belong to mitochondrial quality control which interact with each other to maintain the function and structure of mitochondria. Researchers have attempted to relieve DN by regulating mitochondrial dynamics and mitophagy simultaneously. Mitochondrial fusion protein MFN2 plays a critical role in mitophagy [43,44]. PINK1 could activate MFN2 and then promote Parkin translocation to damaged mitochondria to induce mitophagy [16,45]. The present study showed that MFN2 knockdown inhibited mitophagy induced by DIO through downregulating Parkin and LC3II/LC3I expressions in HK-2 cells. PINK1 knockdown decreased MFN2, Parkin, and LC3II/LC3I expressions then suppressed mitophagy induced by DIO, suggesting that MFN2 plays a critical role in mitophagy induction. Our results also showed that PINK1 could improve mitochondrial dynamics because PINK1 siRNA abolished the improvement effect of DIO on mitochondrial dynamics as evidenced by the increase in DRP1 and *p*-DRP1 expressions and the decrease in MFN1, MFN2, and OPA1 expressions, which may be due to mitophagy inhibition.

## 5. Conclusions

In conclusion, DIO protected against DN through enhancing autophagy and mitophagy by the modulation of the CaMKK2-mediated AMPK-mTOR and PINK1-MFN2-Parkin pathways, respectively. DIO also relieved DN through the improvement of mitochondrial dynamics via targeting CaMKK2. Additionally, there was an interplay between mitophagy and mitochondrial dynamics. On the one hand, MFN2 participated in mitophagy induction; on the other hand, the knockdown of PINK1 counteracted the beneficial effect of DIO on mitochondrial dynamics.

## Figures and Tables

**Table 1:** Physical characteristics and biochemistry parameters in rats at the end of the experiment.

	Control	DN	DN + DIO (10 mg/kg)	DN + DIO (20 mg/kg)	DN + Mef (300 mg/kg)
Fasting blood glucose (mg/dL)	80.1 ± 6.7	490.1 ± 25.3 **	344.3 ± 79.8 ^##^	368.6 ± 90.24 ^##^	346.1 ± 74.5 ^##^
Kidney/body weight (g/100 g)	0.57 ± 0.02	0.89 ± 0.08 **	0.83 ± 0.12	0.83 ± 0.05	0.86 ± 0.08
Urea nitrogen (mg/dL)	26.3 ± 5.6	53.3 ± 8.2 **	37 ± 6.4 ^##^	40.4 ± 7.1 ^##^	29.9 ± 17.4 ^##^
Creatinine (µmol/L)	41.6 ± 5.9	81.9 ± 8.22 **	59.5 ± 9.7 ^##^	56.9 ± 6.8 ^##^	42.5 ± 7.9 ^##^

Data are expressed as mean ± SD (n = 8). ** *p* < 0.01, significantly different compared to the control group; ^##^ *p* < 0.01, significantly different compared to the DN group.

## Data Availability

The data presented in this study are available on request from the corresponding author.

## Supplementary Materials

The following supporting information can be downloaded at: https://www.mdpi.com/article/10.3390/nu15163554/s1, Figure S1: DIO restored autophagy and mitophagy in DN rats; Figure S2: DIO induced autophagy and mitophagy in HK-2 cells exposed to HG; Figure S3: Inhibition of CaMKK2 abolished autophagy and mitophagy induced by DIO in HK-2 cells; Figure S4: DIO improved mitochondrial dynamics in DN rats and in HK-2 cells exposed to HG; Figure S5: The interference efficiency of CaMKK2, PINK1, and MFN2 in HK-2 cells.
