# Supplementary material for: Diosgenin Targets CaMKK2 to Alleviate Type II Diabetic Nephropathy through Improving Autophagy, Mitophagy and Mitochondrial Dynamics
Source: Nutrients. 2023 Aug 11;15(16):3554. doi: 10.3390/nu15163554 (PMC10459415; doi:10.3390/nu15163554)
Supplement: Supplementary file 1 [file nutrients-15-03554-s001.zip › nutrients-2472855-supplementary.pdf]

Supporting information

Diosgenin targets CaMKK2 to alleviate type II diabetic nephropathy through improving autophagy, mitophagy and mitochondrial dynamics

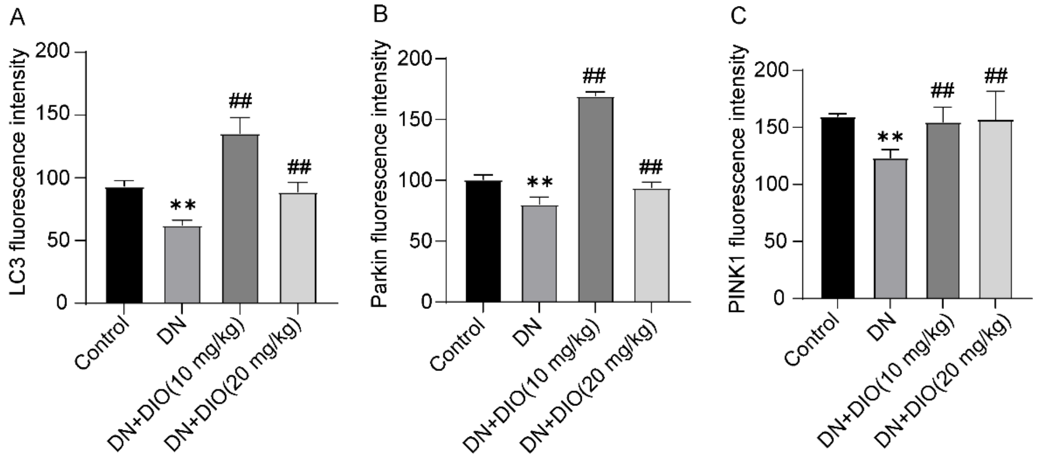

Figure S1. DIO restored autophagy and mitophagy in DN rats. **(A)** Quantification of LC3 immunofluorescence. **(B)** Quantification of Parkin immunofluorescence. **(C)** Quantification of PINK1 immunofluorescence. Data are expressed as mean ± SD, n = 6. *$P < 0.05$ and **$P < 0.01$, significantly different from the Control group; $^{\#}P < 0.05$ and $^{\#\#}P < 0.01$, significantly different from the DN group.

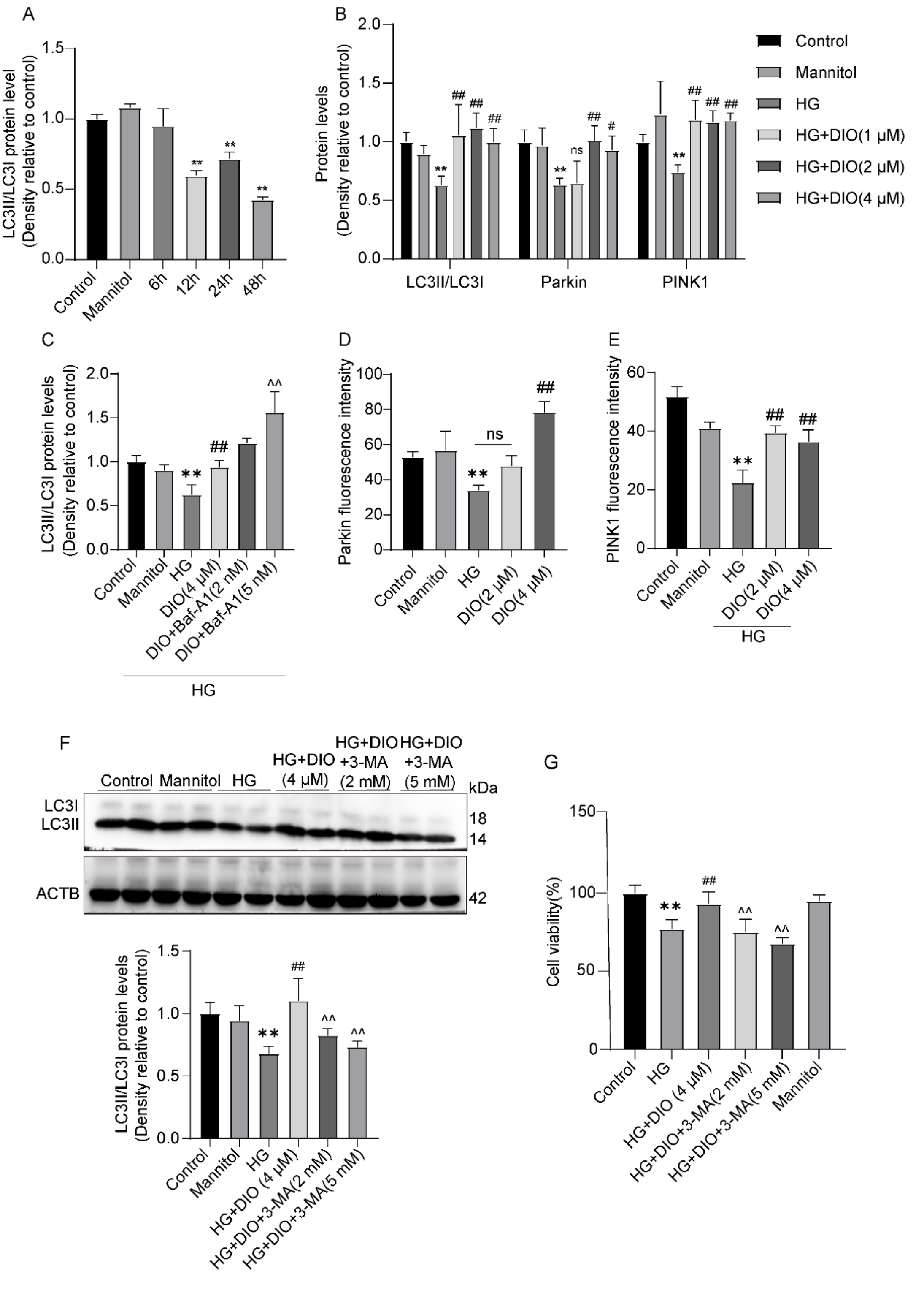

Figure S2. DIO induced autophagy and mitophagy in HK-2 cells exposed to HG. **(A)** Quantification of LC3 protein expression after HG (30 mM) treatment for 6, 12, 24, and 48 h. **(B)** Quantification of LC3, Parkin, and PINK1 protein expressions after DIO treatment. **(C)** Quantification of LC3 protein expression of HG+DIO+Baf-A1 treatment. **(D)** Quantification of Parkin

immunofluorescence after DIO treatment. **(E)** Quantification of PINK1 immunofluorescence after DIO treatment. **(F)** Western blot image and quantification of LC3 expression after 3-MA treatment; **(G)** Cell viability after 3-MA treatment. Data are expressed as mean ± SD, n = 6. $^{*}P < 0.05$ and $^{**}P < 0.01$, significantly different from the Control group; $^{\#}P < 0.05$ and $^{\#\#}P < 0.01$, significantly different from the HG group. $^{\wedge}P < 0.05$ and $^{\wedge\wedge}P < 0.01$ significantly different from HG+DIO group.

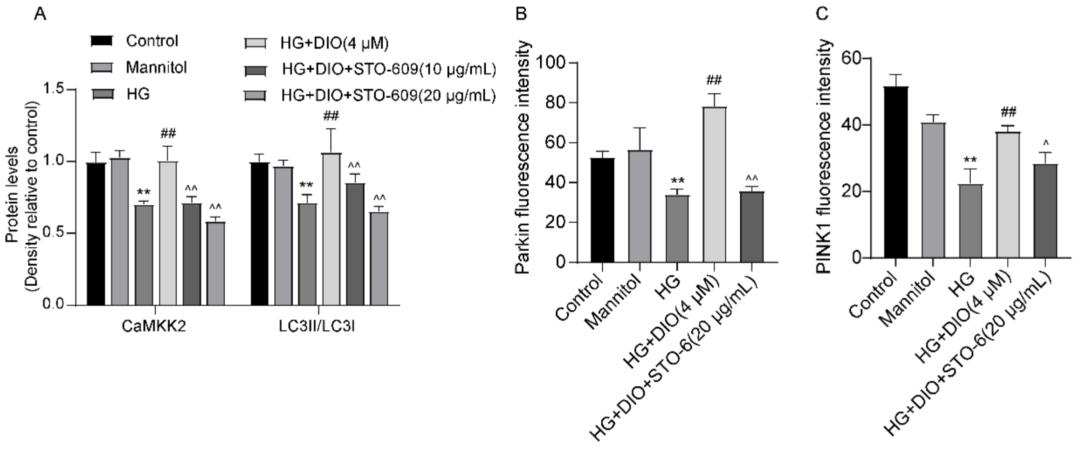

Figure S3. Inhibition of CaMKK2 abolished autophagy and mitophagy induced by DIO in HK-2 cells. **(A)** Quantification of CaMKK2 and LC3 protein expressions. **(B)** Quantification of Parkin immunofluorescence. **(C)** Quantification of PINK1 immunofluorescence. Data are expressed as mean ± SD, n = 6. *$P < 0.05$ and **$P < 0.01$, significantly different from the Control group; $^{\#}P < 0.05$ and $^{\#\#}P < 0.01$, significantly different from the HG group; ^$P < 0.05$ and ^^$P < 0.01$ significantly different from HG+DIO group.

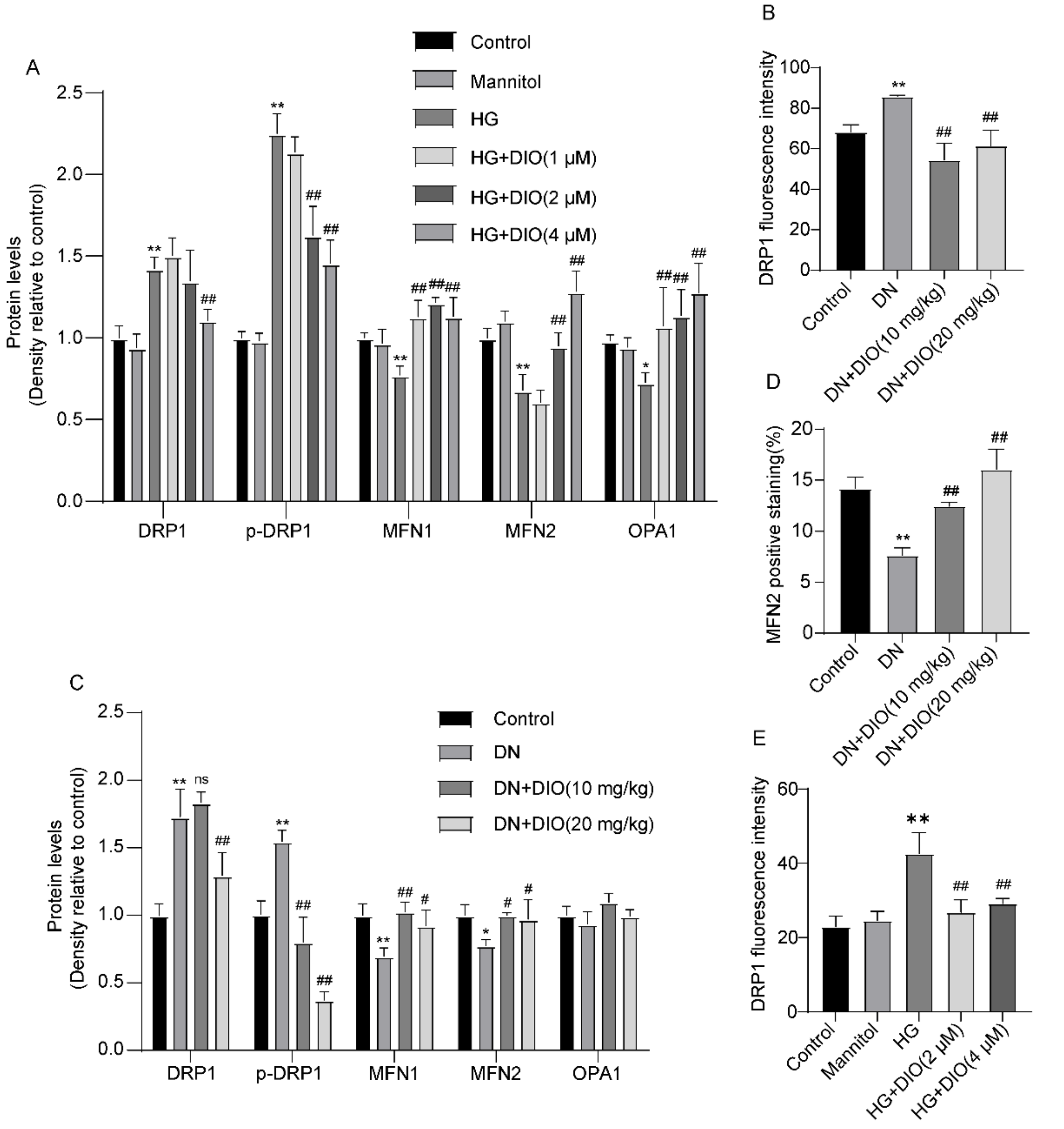

Figure S4. DIO improved mitochondrial dynamics in DN rats and in HK-2 cells exposed to HG. **(A)** Quantification of DRP1, p-DRP1, MFN1, MFN2, and OPA1 protein expressions in HK-2 cells. **(B)** Quantification of DRP1 immunofluorescence in DN rats. **(C)** Quantification of DRP1, p-DRP1, MFN1, MFN2, and OPA1 protein expressions in DN rats. **(D)** Quantification of MFN2 immunohistochemistry in DN rats. **(E)** Quantification of DRP1 immunofluorescence in HK-2 cells. Data are expressed as mean ± SD, n = 6. *$P < 0.05$ and **$P < 0.01$, significantly different from the Control group; $^{\#}P < 0.05$ and $^{\#\#}P < 0.01$, significantly different from the DN or HG group.

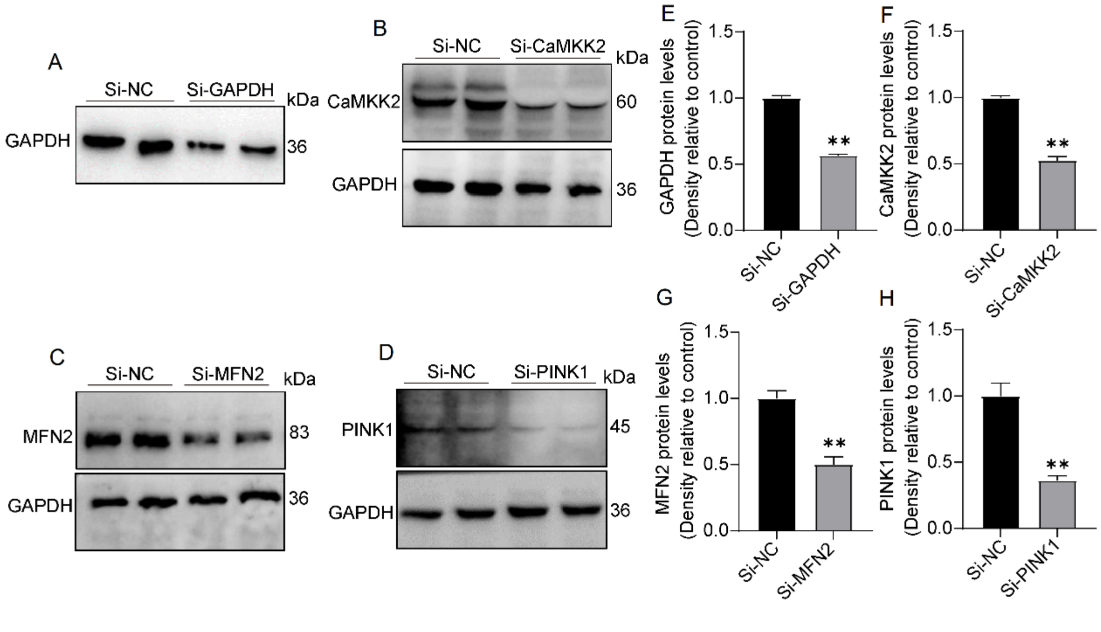

Figure S5. The interference efficiency of CaMKK2, PINK1, and MFN2 in HK-2 cells. **(A)** Western blot image of GAPDH. **(B)** Western blot images of CaMKK2 and GAPDH. **(C)** Western blot images of MFN2 and GAPDH. **(D)** Western blot images of PINK1 and GAPDH. **(E)** Quantification of GAPDH protein expression. **(F)** Quantification of CaMKK2 protein expression. **(G)** Quantification of MFN2 protein expression. **(H)** Quantification of PINK1 protein expression. Data are expressed as mean ± SD, n = 6. *$P < 0.05$ and **$P < 0.01$, significantly different from the Control group.
